# Supplementary material for: HIF-1α Negatively Regulates Irisin Expression Which Involves in Muscle Atrophy Induced by Hypoxia
Source: Int J Mol Sci. 2022 Jan 14;23(2):887. doi: 10.3390/ijms23020887 (PMC8777935; doi:10.3390/ijms23020887)
Supplement: Supplementary file 1 [file ijms-23-00887-s001.zip › ijms-1498244-supplementary.pdf]

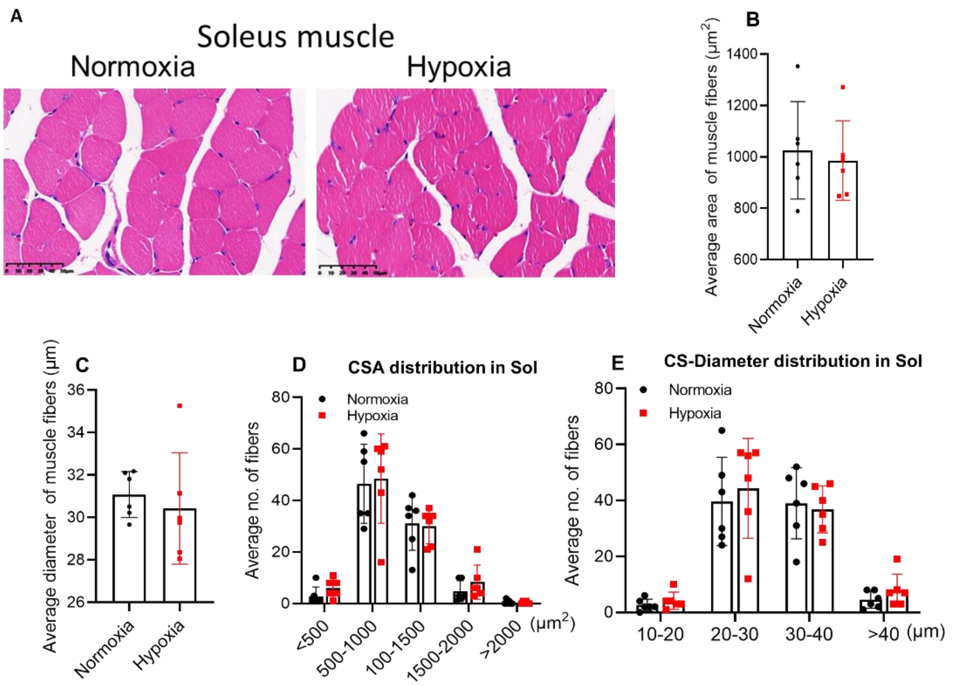

Supplementary Figure S1. The representative images of H&E staining of Sol muscle. Scale bar, 50 μm. (A). Hypoxia has no effect on the average area and diameter (Feret's diameter) (B, C) as well as the distribution of CSA (the cross-sectional area from the mid-belly of Gas muscle) and CS-diameter (D, E) in Sol muscles (n = 6).

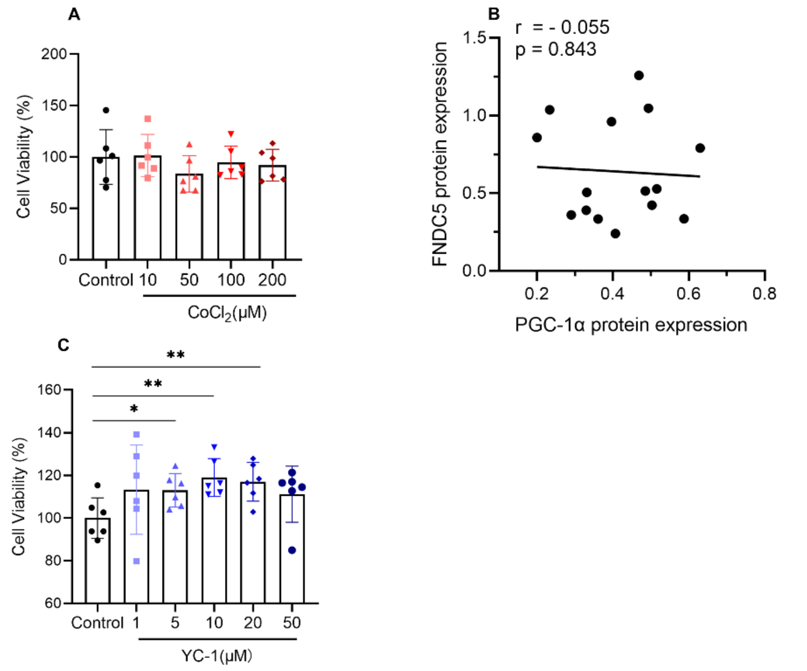

Supplementary Figure S2. Chemical hypoxia induced by $CoCl_2$ treatment (10, 50, 100, and 200 μM) did not affect the viability of C2C12 myotubes (A, n = 6). In hypoxia, the expression of FNDC5 was not correlated with PGC-1*α* (B). Inhibition of HIF-1*α* by YC-1 (1, 5, 10, 20, and 50 μM) mildly affected the viability of C2C12 myotubes (C, n = 6).
